# The Epidemiology, Risk Profiling and Diagnostic Challenges of Nonalcoholic Fatty Liver Disease

**DOI:** 10.3390/medicines6010041

**Published:** 2019-03-18

**Authors:** Umair Iqbal, Brandon J. Perumpail, Daud Akhtar, Donghee Kim, Aijaz Ahmed

**Affiliations:** 1 Department of Medicine, Geisinger Commonwealth School of Medicine, Danville, PA 17822, USA; 2 Department of Medicine, Drexel University College of Medicine, Philadelphia, PA 19129, USA; brandonperumpail@gmail.com; 3 Department of Medicine, University of British Columbia, Vancouver, BC V6T 1Z4, Canada; dakhtar@alumni.ubc.ca; 4 Division of Gastroenterology and Hepatology, Stanford University School of Medicine, Stanford, CA 94305, USA; dhkimmd@stanford.edu (D.K.); aijazahmed@stanford.edu (A.A.)

**Keywords:** nonalcoholic fatty liver disease, nonalcoholic steatohepatitis, NAFLD, NASH, liver biopsy

## Abstract

Nonalcoholic fatty liver disease (NAFLD) encompasses a wide spectrum of liver damage from the more prevalent (75%–80%) and nonprogressive nonalcoholic fatty liver (NAFL) category to its less common and more ominous subset, nonalcoholic steatohepatitis (NASH). NAFLD is now the most common cause of chronic liver disease in the developed world and is a leading indication for liver transplantation in United States (US). The global prevalence of NAFLD is estimated to be 25%, with the lowest prevalence in Africa (13.5%) and highest in the Middle East (31.8%) and South America (30.4%). The increasing incidence of NAFLD has been associated with the global obesity epidemic and manifestation of metabolic complications, including hypertension, diabetes, and dyslipidemia. The rapidly rising healthcare and economic burdens of NAFLD warrant institution of preventative and treatment measures in the high-risk sub-populations in an effort to reduce the morbidity and mortality associated with NAFLD. Genetic, demographic, clinical, and environmental factors may play a role in the pathogenesis of NAFLD. While NAFLD has been linked with various genetic variants, including PNPLA-3, TM6SF2, and FDFT1, environmental factors may predispose individuals to NAFLD as well. NAFLD is more common in older age groups and in men. With regards to ethnicity, in the US, Hispanics have the highest prevalence of NAFLD, followed by Caucasians and then African-Americans. NAFLD is frequently associated with the components of metabolic syndrome, such as type 2 diabetes mellitus (T2DM), obesity, hypertension, and dyslipidemia. Several studies have shown that the adoption of a healthy lifestyle, weight loss, and pro-active management of individual components of metabolic syndrome can help to prevent, retard or reverse NAFLD-related liver damage. Independently, NAFLD increases the risk of premature cardiovascular disease and associated mortality. For this reason, a case can be made for screening of NAFLD to facilitate early diagnosis and to prevent the hepatic and extra-hepatic complications in high risk sub-populations with morbid obesity, diabetes, and other metabolic risk factors.

## 1. Introduction

Nonalcoholic fatty liver disease (NAFLD) is the most common cause of chronic liver disease in the developed world and is one of the leading indications for liver transplantation in the United States (US) [1,2]. Over 64 million people in the US are estimated to have NAFLD, with annual medical costs of approximately $103 billion (1612.18 per patient) [3]. NAFLD comprises nonalcoholic fatty liver (NAFL), which is characterized by the presence of hepatic steatosis without the presence of any substantial inflammation, and nonalcoholic steatohepatitis (NASH), which is defined by the histologic presence of hepatic steatosis with lobular inflammation, and hepatocellular balloon degeneration with and without pericellular fibrosis [3]. The components of metabolic syndrome have been associated with the pathogenesis of NAFLD. These include risk factors such as obesity, diabetes, hypertension, and dyslipidemia [2]. Additionally, the pathogenesis of NAFLD also comprises genetic, demographic, clinical, and environmental factors that all play a role in determining the likelihood of NAFLD. Despite the NAFLD epidemic in chronic liver disease, direct treatment options are still lacking and thus add to the economic burden on healthcare systems [3]. For this reason, there is a need to identify the risk factors associated with NAFLD to effectively screen patients. The purpose of this article is to discuss the epidemiologic trends, risk factors, and the current modalities involved in the diagnostic evaluation of NAFLD. 

## 2. Epidemiology of NAFLD

### 2.1. Prevalence and Distribution of NAFLD

**Figure 1:**
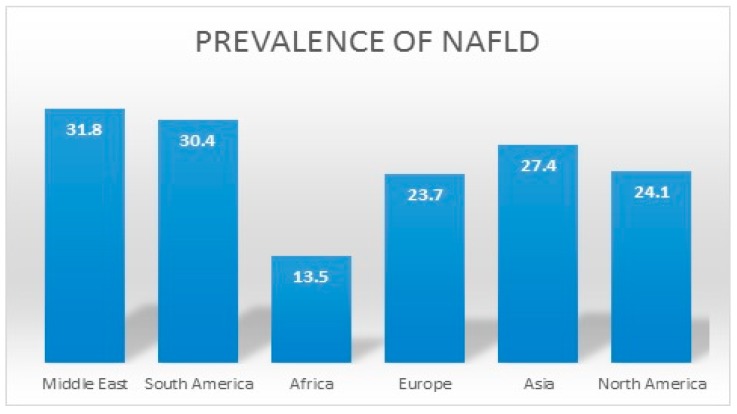
Prevalence of nonalcoholic fatty liver disease (NAFLD) across the world [4].

The worldwide increase in the prevalence of well-established risk factors for NAFLD, such as diabetes, obesity, and age, has had an accompanying increase in the prevalence of NAFLD. The global prevalence of NAFLD is estimated to be around 25% [4]. In a recent meta-analysis, NAFLD was found to be most prevalent in the Middle East (31.8%), followed by South America (30.4%). NAFLD was also found to be least prevalent in Africa (13.5%) [4]. Comparatively, the prevalence of NAFLD in Asia, Europe, and North America was found to be 27.4%, 23.7%, and 24.1% respectively [4]. Figure 1 summarizes the prevalence of NAFLD across the world. The US population has seen a similar trend with the National Health and Nutrition Examination Survey (NHANES), demonstrating a doubling in the prevalence of NAFLD in the US from 5.5% (1988–1994) to 11% (2005–2008) [1]. In a study using the fatty liver index to diagnose NAFLD, the prevalence of NAFLD increased from 18% in 1988–1991 to 31% in 2011–2012 [5]. Concerningly, the prevalence of NASH in the general population is estimated to range between 1.5% and 6.4% [4]. This increasing prevalence of NAFLD in the US parallels the increase in prevalence of NAFLD-related risk factors, which include insulin resistance, obesity, hypertension, and dyslipidemia.

### 2.2. Populations with NAFLD

Diabetes and obesity, in particular, are significant health concerns that are heavily associated with NAFLD. A recent systematic review estimated the global prevalence of NAFLD in diabetic patients to be around 58% [6]. Specifically, the prevalence of NASH in diabetic patients has been demonstrated to be close to 65% [6,7]. Furthermore, NAFLD is also highly prevalent in the overweight and obese population. The prevalence of NAFLD in morbidly obese patients who undergo bariatric surgery has been reported to be as high as 95% [8]. Although there is a strong association between NAFLD and excess body weight, NAFLD can also present in patients with a normal body mass index (BMI). Approximately 5–10% of NAFLD patients in the US are within the normal limits for BMI and are commonly referred to as having lean NAFLD [9]. The pathogenesis of lean NAFLD is analogous to that of obese NAFLD and is similarly accompanied by excessive abdominal fat, diabetes, and hypertension. Age is another important consideration with regard to NAFLD. NAFLD is more prevalent in older patients, with the majority of patients being diagnosed between 40 to 50 years of age. The risk of NASH and fibrosis also increases with age. The prevalence of NAFLD also varies with gender and ethnicity, with there being a greater prevalence in males and individuals of Hispanic descent [4]. Yang et al. displayed that men are at a higher risk of severe fibrosis when compared to premenopausal females. The risk, however, is similar in post-menopausal females, suggesting that female hormones may have protective effects on hepatic fibrosis [10]. A remodeling study by Estes et al. estimated that the prevalence of NAFLD and NASH in the US will rise by 21% and 63%, respectively, with a correlated increase of 178% in liver-related mortalities to an estimated 78,300 deaths by 2030. For this reason, there is a critical need to identify and treat potentially modifiable risk factors in order to reduce the morbidity and mortality associated with this serious disease [11].

## 3. Risk Profiling for NAFLD

### 3.1. Genetic Predisposition to NAFLD

Although obesity, metabolic syndrome, and insulin resistance are the most prevalent risk factors that lead to the development of NAFLD, NAFLD varies substantially among subjects with comparable lifestyles, environmental exposures, and metabolic abnormalities. This suggests that other factors do contribute to the pathogenesis of NAFLD, with genetic predisposition possibly being an important factor. Several epidemiological, familial, and twin studies have shown possible familial predispositions for NAFLD. Struben et al. demonstrated in a study of eighteen members the coexistence of histologically proven NASH and cirrhosis among multiple generations of kindreds [12]. A family study of 157 individuals with familial combined hyperlipidemia revealed an increased prevalence of fatty liver and elevated levels of ALT in both dyslipidemic patients and family members with normal lipid levels [13]. Loomba et al. further revealed a strong association between serum GGT levels, fatty liver, and beta-2-adrenergic receptors in identical twins [14]. NAFLD has been shown to cluster in families with certain genetic variants. These include TM6SF2, PNPLA3, NCAN, and PPP1R3B genes that increase the heritability of NAFLD to up to 27% [15,16,17,18,19,20,21]. One genetic variant linked with NAFLD is a missense mutation [Ile148->Met148 (I148M)] in the Palatin-like phospholipase domain-containing 3 gene (PNPLA3) [9]. PNPLA3 exerts a strong influence on hepatic fat accumulation in GG homozygous individuals, showing 73% more hepatic fat content when compared with CC homozygous individuals. These patients were also more susceptible to the development of more severe histologic liver damage, with a 3.24-fold greater risk of higher necro-inflammatory scores and a 3.2-fold greater risk of developing fibrosis [15]. These associations were maintained irrespective of the degree of obesity or the presence of diabetes [16,17]. The single variant in the PNPLA3 gene (I148M) has been shown to occur with the greatest frequency in Hispanics, followed by non-Hispanic whites, and the least in African Americans [9]. This may explain the low prevalence of NAFLD in African Americans despite the higher prevalence of obesity and diabetes in this population [4]. A minor allele in transmembrane 5 superfamily member 2 (*TM6SF2)* was associated with magnetic resonance spectroscopy-measured hepatic triglyceride content from the Dallas Heart Study [20]. In addition, the minor allele of *TM6SF2* was noted to increase the risk for hepatic fibrosis independent of age, obesity, diabetes, and *PNPLA3* genotype [21]. Other genetic variants, such as Farnesyl diphosphate farnesyl transferase I (FDFT1), Collage type XIII, alpha 1 (COL13A1), and neurocan (NCAN), which have been identified by genomewide scans, have also been implicated with increased susceptibility to NAFLD [19]. Table 1 summarizes these different genetic variants linked with NAFLD.

### 3.2. Gender and Age-Related Risk for NAFLD

Gender and age are important considerations with regards to NAFLD. The prevalence of NAFLD and NASH is generally higher in men [22,23,24,25]. However, some studies have also reported a higher prevalence of NAFLD in women [26,27]. In men, NAFLD follows an “Inverted U-Shaped Curve” with the prevalence increasing from younger to middle-aged individuals and then decreasing around the age of 50 [23,24,25]. The prevalence of NAFLD appears to be lower in premenopausal women and increases significantly after the age of 50, ultimately, peaking in the 6th decade of life [23,24,25]. Yang et al. displayed that men are at a higher risk of severe fibrosis when compared to premenopausal women. The risk, however, is similar in post-menopausal women, suggesting that female hormones may have protective effects on hepatic fibrosis [10]. NAFLD is more prevalent with advanced age due to an increase in the prevalence of NAFLD-related risk factors, such as metabolic syndrome, diabetes, and hypertension. The risk of fibrosis and NASH also increases with advancing age. A recent systematic review demonstrated that advancing age was found to be an independent predictor of advanced fibrosis [28]. Advancing age also increases the extrahepatic manifestations of NAFLD and, thus, there is an increased risk for morbidity and mortality in older populations. The effects of gender on the outcomes of NAFLD is still unclear, although some studies have reported worse outcomes of NAFLD in men [29,30]. 

### 3.3. Differences in NAFLD from Race/Ethnicity

Different ethnic groups display multiple differences with regards to NAFLD when pertaining to its prevalence, gender distribution, and genetic predisposition. Specifically, the prevalence of NAFLD and NASH varies in individuals with different ethnicities. Hispanics exhibit the greatest prevalence of NAFLD, which is then followed by Caucasians [4,8]. Interestingly, African-Americans have the lowest prevalence of NAFLD despite having the highest prevalence of obesity and metabolic syndrome [8]. Additionally, the prevalence of NASH is also less prevalent in African-American patients with NAFLD when compared to Hispanic patients. It should be noted, though, that the prevalence of advanced fibrosis did not differ [31]. Different ethnicities also display gender differences in the distribution of NAFLD. NAFLD is more prevalent in Caucasian men when compared to Caucasian women. This pattern is not seen in the Hispanic and African-American populations [32]. Certain genetic variants, such as the I148M variant of PNPLA3, also differ between ethnicities. I148M, which is strongly associated with hepatic fat content and NAFLD, is highly frequent in Hispanics (49%) in comparison to non-Hispanic Caucasians (23%) and African-Americans (17%) [18]. Studies have also demonstrated an increased prevalence of I148M in Indian Asians, which may explain the high prevalence of NAFLD in these populations [33]. The literature is still unclear regarding outcomes in NAFLD pertaining to the progression of cirrhosis and mortality in these different ethnic groups. For this reason, more research is warranted to further delineate the role of ethnicity and genetic predisposition in NAFLD-related outcomes. Table 2 summarizes these nonmodifiable risk factors of NAFLD.

### 3.4. Excessive Abdominal Adipose Tissue Deposition and Obesity

Obesity is a major modifiable risk factor for NAFLD, with over one third of the US population struggling with obesity and the remaining two thirds being overweight [34]. NAFLD is two times more prevalent in overweight individuals and four times more prevalent in the obese population. Weight gain is strongly associated with the development of NAFLD, with even a modest weight gain demonstrating an increase in the risk of developing NAFLD [35,36]. Obesity also increases the risk of NASH, fibrosis, and hepatocellular carcinoma, but current data are conflicting with some studies, failing to show an association between obesity and the progression of fibrosis [37,38]. The distribution of adipose tissue is more clearly associated to NAFLD than the amount of adipose tissue as presenting evidence displays a strong link between visceral adiposity and NAFLD. Visceral adiposity is associated with insulin resistance and increased hepatic fat content. The release of pro-inflammatory and pro-fibrogenic mediators, such as tumor necrotic factor (TNF) and leptin by visceral fat tissue, may play a role in the increased risk of fibrosis. By contrast, subcutaneous adiposity is inversely related to hepatic fat content [39,40]. Asian populations depict a greater occurrence of visceral adipose tissue, which predisposes them to NAFLD at a lower body mass index (BMI) [41]. A recent meta-analysis revealed that obesity (as per ethnic-specific BMI cutoff) could predict a worse long-term prognosis in patients with NAFLD [42]. Although there is a strong association between NAFLD and obesity, there is a growing number of patients with NAFLD that have a normal BMI and are referred to as having lean NAFLD. Lean NAFLD comprises 5–10% of patients with NAFLD in the US [9]. The prevalence of lean NAFLD is higher in Europeans and Asians [43,44]. These patients share common risk factors with obese NAFLD, with the increased prevalence of risk factors, such as hypertension and type 2 diabetes mellitus (T2DM). Obesity also increases the risk for cardiovascular diseases. Therefore, efforts should be made to adopt healthy lifestyles and weight loss in order to prevent the morbidity and mortality associated with NAFLD.

### 3.5. Insulin Resistance and Diabetes as a Risk Factor for NALFD 

Insulin resistance plays a major role in the pathogenesis of NAFLD. Adipose tissue releases pro-inflammatory cytokines, which is associated with insulin resistance. There are several other potential mechanisms of insulin resistance in NAFLD, which include increased lipogenesis, mitochondrial fatty acid oxidation, serum free fatty acids (FFA) levels, and adiponectin [45]. Patients with NAFLD have higher levels of FFA and lower levels of serum adiponectin. There is also a potential role of intestinal microbiota in insulin resistance, as composition of intestinal microbiota is distinct in patients with NAFLD patients compared to non-NAFLD patient population [45,46].

The prevalence of NAFLD is significantly higher in patients with T2DM and ranges from 30% to 70% in various population-based studies. Overweight and obese individuals with T2DM are at an even higher risk for NAFLD. NAFLD has been reported to be as high as 76% and 56% in obese T2DM patients and biopsy-proven NASH, respectively [47]. A recent systematic review estimated the overall global prevalence of NAFLD to be around 58% in T2DM patients [6]. Although a correlation exists between hepatic fat content and aminotransferase levels, most T2DM patients have normal serum aminotransferase levels despite having higher hepatic fat content. For this reason, T2DM can have NASH and fibrosis with normal liver chemistries [48]. T2DM patients are also at a higher risk for developing NASH and liver-related complications, including mortality related to cirrhosis [49,50]. Adams et al. revealed a two-fold increased risk of all-cause mortality in T2DM patients [51]. A study by Zoppini et al. revealed similar findings with a three- to five-fold higher mortality mostly related to NAFLD [52]. NAFLD is also associated with cardiovascular disease, which is highly prevalent in T2DM, and recent studies demonstrate that NAFLD patients may have a higher risk of developing microvascular complications of T2DM [53,54]. These findings suggest that screening for NAFLD should be considered in T2DM, as the presence of NASH can influence the treatment choice of T2DM. Insulin sensitizers have shown to be beneficial in improving the metabolic derangements in patients with NAFLD/NASH [55]. Multiple studies have shown the benefit of pioglitazone in improving the biochemical and histological parameters of NASH [56,57]. AASLD guidelines also recommend considering the use of pioglitazone in biopsy-proven NASH patients [58]. Liraglutide has also been studied in NASH patients, and early investigations show some benefits. This does require further research to prove its clinical efficacy in the treatment of NASH [59]. Although several studies have been done on evaluating the efficacy of metformin on liver chemistries and histology, AASLD guidelines do not recommend the use of metformin in NASH, as it has not been shown to improve liver histology [58].

### 3.6. Dyslipidemia

Lipotoxicity can occur secondary to the accumulation of fat content in non-adipose tissues and can occur secondary to insulin resistance in patients with NAFLD. It plays a pivotal role in 5h3 progression of a milder form of NAFLD to NASH, as patients with NAFLD have higher levels of triglycerides, FFAs, and other types of lipids, like bile acids, free cholesterol, lysophosphatidyl cholines, and ceramides [60]. As described above, elevated levels of FFAs can also promote insulin resistance and increase the risk of NAFLD [45,60]. Hypertriglyceridemia (HTG) is also an independent predictor of NAFLD, and screening for NAFLD should be considered in this patient population, especially in the context of elevated liver enzymes [61,62]. Proprotein convertase subtilisin/kexin type 9 (PCSK9) is released by hepatocytes and inhibits the uptake of low-density lipoproteins (LDL). PCSK9 levels also increase with hepatic fat accumulation and are associated with a degree of steatosis [63]. 

Risk factors for HTG are similar to those of NAFLD and include insulin resistance and sedentary lifestyle. HTG also increases the risk of cirrhosis [64]. Studies have also shown an association between NASH and elevated levels of non-HDL cholesterol, which also increases the risk of cardiovascular diseases. Kantartzis et al., in a study of 16 patients with fatty liver and 24 control subjects, revealed that fatty liver is significantly associated with lower levels of high density [65]. Similar results were reported from the community-based Framingham Heart study, which revealed that patients with fatty liver had a higher prevalence of HTG and low HDL levels [66]. Given the association of dyslipidemia with NAFLD, several anti-hyperlipidemic drugs have been studied and have been shown to be effective in improving the histological features of NASH. Statins are the most studied drug and have been shown to prevent hepatic fibrosis. Additionally, statins also decrease the risk for cardiovascular diseases, which are highly prevalent in NAFLD patients. A meta-analysis of 259,453 patient showed that statins can decrease the progression of hepatic fibrosis, prevent hepatic decompensation in cirrhosis, and reduce all-cause mortality in patients with chronic liver disease [67]. In addition, statins have been shown to have anticancer properties and may prevent the development of hepatocellular carcinoma (HCC) [68]. Therefore, statins should be considered for primary or secondary prevention of cardiovascular diseases, reducing risk of cirrhosis and HCC in patients with NAFLD/NASH [69,70].

### 3.7. Intestinal Microbiota and Oxidative Stress

Alteration in intestinal microbiota has been shown to play a role in the development of NAFLD. Patients with NAFLD have a higher prevalence of small intestinal bacterial overgrowth and elevated levels of tumor necrosis factor-alpha (TNF-alpha) [46,71]. A study performed on 57 patients with biopsy-proven NASH revealed that abundance of bacteroides was an independent risk factor of NASH. One possible mechanism of injury induced by intestinal microbiota is by endotoxin production, which increases the risk of steatosis [63]. Rifaximin is a minimally absorbable antibiotic, which has been shown to be effective in patients with NAFLD by improving liver enzymes and decreasing NAFLD fat score. Further research is ongoing, studying the role of intestinal microbiota in the pathogenesis of NAFLD [72]. Oxidative stress has also been shown to be a significant risk factor of NAFLD. Insulin resistance can result in hyperinsulinemia, which in turn can block the mitochondrial oxidation of fatty acids, which are then partially metabolized by the peroxisomes and microsomes, with the subsequent production of reactive oxidation species (ROS) and lipid peroxidation [73]. This production of ROS and lipid peroxidation can exhaust antioxidant enzymes and make hepatocytes susceptible to injury [74]. Several clinical studies have shown elevated oxidative stress and lipid peroxidation in patients with NAFLD [73,74]. Therefore, antioxidants can play a therapeutic role in improving the metabolic parameters in patients with NAFLD. Vitamin E is a potent antioxidant which has been shown to be effective in patients with NAFLD/NASH. In a randomized controlled trial of 247 patients, use of vitamin E was associated with improvement in nonalcoholic steatohepatitis compared to placebo [57].

### 3.8. Metabolic Syndrome

The definition of metabolic syndrome is controversial and includes risk factors for cardiovascular diseases such as hypertension, insulin resistance, obesity, and dyslipidemia [4]. There is a strong association between NAFLD and the presence of metabolic syndrome. In a meta-analysis of 411 patients, hypertension increased the risk of developing of hepatic fibrosis [38]. NAFLD has also been shown to increase the risk of arterial hypertension. A prospective cohort study of 22,090 Korean men revealed that the incidence of hypertension increases with the degree of NAFLD and also illustrated that NAFLD is an independent predictor of hypertension [50,75]. Although studies have shown some association between hypertension and NAFLD, the evidence is weaker when comparing the association of NAFLD with diabetes and obesity. Ultimately, the screening of NAFLD should still be considered in hypertensive patients due to the higher prevalence of NAFLD in this population. Table 3 summarizes major modifiable risk factor associated with NAFLD.

Although the components of metabolic syndrome, such as obesity, diabetes, and dyslipidemia, constitute the major risk factors associated with NAFLD, there are several other possible risk factors described in the literature. There is evidence which suggests that macro- and micro-nutrients may contribute to the epidemic of NAFLD. Specifically, increased fructose consumption has been shown to increase the risk of NAFLD [76]. Fructose is not only a major contributor in development of hepatic steatosis but is a risk factor for promoting progression to NASH [77]. It is thought to be secondary to de novo lipogenesis and inflammatory responses promoted by increasing blood glucose levels that lead to hepatocyte apoptosis via the c-Jun-N-Terminal (JNK) pathway [78]. The consumption of beverages with high sugar content has increased five-fold in the US since 1950—a concerning trend. There is evidence linking regular intake of servings with sugar-containing beverages for 6 months to features consistent with NAFLD [79]. Omega-3-fatty acids has also been shown to be effective in reducing hepatic fat accumulation in patients with NAFLD. A meta-analysis including 10 randomized controlled trials with 577 patients of NAFLD revealed Omega-3-fatty acids to be effective in reducing hepatic fat content in patients with NAFLD and NASH [80]. The Mediterranean diet has also been shown to be beneficial in patients with NAFLD. The Mediterranean diet consists of fruits, vegetables, olive oil, and nuts, which are high in omega-3-fatty acids and antioxidants. Baratta et al., in a study including 584 patients, revealed adherence to the Mediterranean diet was associated with lower insulin resistance and reduced risk of NAFLD [81]. Therefore, experts recommend the use of the Mediterranean diet for weight loss and protection against NAFLD [82,83]. There are several other micro- and macronutrients that may influence the development of NAFLD, but detailed discussion of the nutrients is beyond the scope of this review. Other comorbidities, such as hypothyroidism, hypopituitarism, polycystic ovarian disease, and obstructive sleep apnea, that are also associated with NAFLD require further investigations to strengthen their associations [58].

## 4. Diagnostic Challenges in NAFLD

The diagnosis of NAFLD involves an appropriate clinical history, radiographic and laboratory investigations, and histologic information. Diagnosing NAFLD requires evidence of hepatic steatosis in the absence of significant alcohol consumption, other causes of hepatic steatosis, and coexisting liver diseases. The use of abdominal imaging can be used to diagnose NAFLD, thus limiting the need for invasive procedures, such as liver biopsies. However, a liver biopsy is useful when differentiating between simple steatosis (NAFL) and NASH. For this reason, a liver biopsy can assist with determining the risk of disease progression and subsequent management of NAFLD [58,84]. 

### 4.1. Role of Liver Biopsy in the Diagnosis of NAFLD

The early diagnosis of NASH has crucial management implications, as patients can benefit from newly approved medications and off-label therapies to retard the progression of liver disease [57,67]. Unfortunately, a liver biopsy is needed to confirm the characteristic histologic features of NASH prior to making a diagnosis. For this reason, the considerable disease burden of NASH in the US and the invasive nature of a liver biopsy has prompted experts to recommend its selective use only in NAFLD patients with higher likelihoods of progression to NASH. Therefore, an individualized assessment considering the risks and benefits regarding a diagnostic liver biopsy is warranted. Generally, a liver biopsy is reserved for scenarios where the clinical suspicion of NAFLD is high but noninvasive modalities are inconclusive, where there is evidence of cirrhosis or in patients who are at a high risk for advanced fibrosis. The NAFLD activity score (NAS) is widely used for the histopathological diagnosis of NASH [58]. The NAS score considers three important histological features in a biopsy specimen which include steatosis, hepatocellular ballooning, and lobular inflammation. Table 4 describes the NAS score for the diagnosis of NASH. The CRN scoring system is widely used to assess the extent of fibrosis in biopsy specimens in patients with NAFLD [84]. Table 5 describes the CRN histological scoring system for the diagnosis of NAFLD. An important consideration is inter-observer variability that can occur during the histologic evaluation of liver biopsy samples even among experienced pathologists [84,85]. The lack of agreement between pathologists regarding hepatocellular ballooning or sampling error is a limitation and may explain the lower number of patients meeting the entry criteria for clinical trials. 

### 4.2. Noninvasive Markers for the Detection of Fibrosis

The limitations of liver biopsy, the high prevalence of NAFLD, and the lack of agreement on the clinical predictors of NASH have created a niche for next-generation noninvasive biomarkers and imaging modalities to help to differentiate NAFLD from NASH. These biomarkers can be categorized into indirect and direct markers of fibrosis, as illustrated in Table 6. Some examples of indirect markers include aminotransferases, cytokeratin-18, and multiple scoring systems which combine certain laboratory markers, such as the aspartate aminotransferase (AST)/platelet ratio index (APRI), NAFLD Fibrosis Score (NFS), FIB-4 index, and Fibrotest. Direct markers of fibrosis comprise the extracellular matrix (ECM). such as hyaluronic acid (HA), fibronectin, elastin, and laminin, which form in the setting of persistent hepatocyte injury and have also been incorporated into certain scores. These markers are discussed in detail below. 

### 4.3. Indirect Markers of Fibrosis

#### 4.3.1. Aminotransferases

Elevations of alanine aminotransferase levels (ALT) and aspartate aminotransferases (AST) can be found in patients with NAFLD, although normal levels do not exclude the presence of NAFLD or NASH. When elevated, these patients usually have AST to ALT ratios of less than 1, unlike alcoholic liver disease, which typically demonstrates ratios greater than 2. A retrospective study by Verma et al. reported the prevalence of NASH in patients with elevated ALT levels (>35 U/L) to be 28.9% compared to the normal ALT group, which had a frequency close to 10.7%. Additionally, there was no difference in the rate of advanced fibrosis between the normal and elevated ALT group. The study also revealed that ALT levels two times the upper limit of normal have limited utility in predicting NASH, with a sensitivity and specificity of 50% and 61%, respectively [86].

#### 4.3.2. Cytokeratin-18

Multiple studies have found elevated levels of cytokeratin-18 (CK-18) fragments in patients with NASH and have shown that elevated levels of CK-18 can be a predictor of NASH in patients with NAFLD [87]. Although promising, the commercial unavailability of these tests and the lack of a clear cutoff point are factors which limit its clinical use in the diagnosis of NASH. Further studies are required to further establish the clinical utility of CK-18 in NAFLD.

#### 4.3.3. AST/Platelet Ratio Index (APRI)

APRI is a ratio of ALT levels and platelets that has been used to predict advance fibrosis primarily in HIV and HCV patients with limited evidence in the NAFLD population. A meta-analysis including 40 studies showed that the APRI ratio had a sensitivity and specificity of 77% and 72%, respectively, when predicting for significant fibrosis [88]. 

#### 4.3.4. NAFLD Fibrosis Score

The NAFLD fibrosis score (NFS) is a score used to evaluate the possibility of advanced fibrosis in patients with NAFLD and is also used to predict outcomes in patients with NAFLD. NFS considers a patient’s age, BMI, blood glucose levels, aminotransferase levels, platelet count, and albumin. In a validation study, a high NAFLD fibrosis score cutoff (>0.676) was associated with a positive predictive value of 90 percent, and a low cutoff value (<−1.455) was associated with a negative predictive value of 93 percent [89]. A retrospective study by Angulo et al. in patients with NAFLD revealed the area under the ROC curve using NFS was 0.86 for predicting adverse liver-related outcomes and 0.70 for predicting mortality or liver transplantation [90].

#### 4.3.5. FIB-4 Index

FIB-4 incorporates a patient’s age, AST, ALT, and platelet count to evaluate advanced fibrosis. FIB-4 has been shown to be better at predicting advanced fibrosis when compared to other serologic tests [91]. FIB-4 is also helpful in predicting outcomes in patients with NAFLD. The previous retrospective study by Angulo et al. further evaluated the FIB-4 index and revealed the area under the ROC curve was 0.81 for predicting adverse liver-related outcomes and 0.67 for predicting death or liver transplantation [90].

#### 4.3.6. FibroTest, FibroSure, and ActiTest

FibroTest includes the assessment of a patient’s age, sex, alpha-2-macroglobulin, haptoglobin, gamma globulin, apolipoprotein A1, gamma glutamyl transferase, and total bilirubin levels to predict the extent of fibrosis [92,93]. ActiTest is similar to FibroTest, but it incorporates ALT levels [93]. A meta-analysis including 1570 patients revealed that these noninvasive proprietary tests were reliable alternatives to liver biopsy in patients with chronic HCV [94]. 

### 4.4. Direct Markers for Fibrosis

The liver is capable of hepatic regeneration when injured. When exposed to severe insults, however, hepatocytes can be replaced by components of the extracellular matrix (ECM), such as hyaluronic acid (HA), fibronectin, elastin, and laminin. These markers of ECM are attractive targets to predict fibrosis in NAFLD patients and are currently under investigation [95,96,97].

#### 4.4.1. Enhanced Liver Fibrosis panel (ELF)

ELF is a commercially available panel that incorporates matrix turnover markers, such as matrix metalloproteinase 1 (MMP-1), HA, and amino-terminal propeptide of type III collagen level to predict liver fibrosis [95]. ELF is marginally better than NFS in predicting fibrosis, but the combination of these two tests has been shown to remarkably enhance the detection of fibrosis [96].

#### 4.4.2. FibroSpect II

The FibroSpect II panel consists of serum HA, tissue inhibitor of metalloproteinase-1 (TIMP-1), and alpha-2-macroglobulin. In a retrospective of study of 129 morbidly obese patients undergoing gastric bypass, Fibrospect II had a negative predictive value of 100% to predict advance fibrosis and can be used as a useful panel to rule out significant fibrosis in NAFLD patients [97].

### 4.5. Abdominal Imaging Modalities for Assessing Hepatic Steatosis

A variety of imaging modalities can be utilized for the diagnosis of NAFLD as displayed in Table 7. These range from common radiologic techniques, such as ultrasound (US), computed tomography (CT), and magnetic resonance imaging (MRI), to methods measuring hepatic stiffness, such as magnetic resonance elastography (MRE) and Transient elastography (TE) (Fibroscan). 

#### 4.5.1. Ultrasound and Computed Tomography of the Abdomen

US of the abdomen is the most widely available test, with a meta-analysis reporting its sensitivity and specificity for diagnosing fatty liver disease as high as 85% and 94% when compared to liver biopsy [98]. Additionally, US is a noninvasive modality that does not pose contrast-related risks and is preferred by patients over more invasive methods. US is, however, operator-dependent and thus lacks sensitivity in NAFLD patients with less than 30% steatosis on liver biopsy. Sensitivity of US in detecting hepatic steatosis also decreases in morbidly obese patients. Computed tomography (CT) is another imaging modality but requires radiation and contrast exposure, has lower sensitivity for hepatic fat mapping, and is expensive [8]. 

#### 4.5.2. Magnetic Resonance Imaging and Magnetic Resonance Spectroscopy

Magnetic resonance imaging (MRI) and magnetic resonance spectroscopy (MRS) provide the highest precision (sensitivity and specificity) in quantifying steatosis and liver fat mapping when compared to other imaging modalities. With promising initial data, MRI and MRS may become the gold standard for the diagnosis and management of NAFLD in the near future, although the cost of such imaging is still a significant health and economic barrier [99,100,101]. 

Alternatively, hepatic stiffness measurements with noninvasive methods like MRE and TE can also be used to stratify patients into advanced and nonadvanced fibrosis groups [102,104,105,107]. 

#### 4.5.3. Transient Elastography (Fibroscan) and Magnetic Resonance Elastography (MRE)

Fibroscan and MRE are the most accurate noninvasive tests to evaluate hepatic stiffness and to differentiate advanced and nonadvanced hepatic fibrosis. Fibroscan has a sensitivity of 88%, with a negative predictive value of 90% in detecting advanced fibrosis [102]. Comparatively, MRE has a sensitivity of 86% and specificity of 91% for diagnosing advanced fibrosis by using a stiffness cutoff of 3.63 Kilopascals [104]. MRE can also help to identify individuals with steatohepatitis, even before the onset of significant fibrosis. NAFLD with inflammation but without fibrosis demonstrates greater hepatic stiffness than simple steatosis and a lower mean stiffness than NAFLD with fibrosis [107]. MRE is also superior to Fibroscan in diagnosing cirrhosis [105]. Limitations for using MRE include limited availability and expertise to interpret the results, cost of the procedure, inability to be used in the presence of metal implants, and patient-limiting factors, such as size and claustrophobia [103]. Although Fibroscan is more convenient to use, its clinical utility is limited in patients with ascites, excessive abdominal fat disposition or in the presence of acute inflammation [106]. For these reasons, Fibroscan is a more appropriate modality in non-obese patients, while MRE should be considered in patients with morbid obesity for the detection of advanced fibrosis.

In summary, the diagnosis of NAFLD can be made by combining appropriate noninvasive serologic tests and imaging modalities and may help in reducing the need for liver biopsies. Studies have shown that these noninvasive markers of liver fibrosis can differentiate between the absence of fibrosis and mild fibrosis from advanced bridging fibrosis or cirrhosis. However, noninvasive tests lack the ability to reliably detect intermediate or moderate grade fibrosis and are not useful in staging the extent of hepatic injury. For this reason, liver biopsy remains the gold standard test when diagnosing NAFLD and when evaluating advanced fibrosis and should be considered when the diagnosis remains unclear despite noninvasive testing.

## 5. Conclusions

NAFLD is a complex entity that overlaps significantly with insulin resistance and inflammatory states, including the components of metabolic syndrome. Furthermore, there is emerging evidence highlighting the importance of genetic predisposition, environmental exposures, clinical factors, and demographical differences in NAFLD. Several studies illustrate the importance of nonpharmacologic and pharmacologic measures in reducing the incidence and progression of NAFLD. The need for early intervention can only be stressed further with evidence linking NALD with increased cardiovascular mortality. For these reasons, efforts should be made to effectively screen and treat for NAFLD and its associated risk factors to impede the progression of this serious disease, which currently lacks a definite treatment.

## Figures and Tables

**Table 1:** Genetic variants associated with NAFLD.

Gene	Effect on NAFLD/NASH
Palatine like phospholipase domain containing 3 (PNPLA-3) [15,16,17]	Increased risk of hepatic steatosis, incidence of NASH and severity of fibrosis
Farnesyl diphosphate farnesyl transferase I (FDFT1) [19]	Increased severity of NAFLD activity score
Collagen type XIII alpha 1 (COL13A1) [19]	Increased severity of fibrosis
Neurocan (NCAN) [19]	Increased risk hepatic steatosis
Glucokinase regulatory protein (GCKR) [19]	Increased risk hepatic steatosis
Transmembrane 5 superfamily member 2 (TM6SF2) [21]	Increased risk for hepatic fibrosis

**Table 2:** Nonmodifiable risk factors for NAFLD.

Factor	Association with NAFLD	Risk of Progression to NASH
Age [23,24,25,28]	Increases prevalence with age	Increase risk of progression to NASH
Gender [22,23,24,25]	Increase prevalence in men	Unclear
Ethnicity [8,18,31,33]	Hispanics has the highest prevalence followed by Caucasians and African-American	Association is still uncertain. African-American has less prevalence of NASH despite highest prevalence of obesity.
Familial predisposition [12,14]	Family history of NAFLD might increases the risk	Unclear

**Table 3:** Modifiable risk factors for NAFLD.

Factor	Association with NAFLD	Risk of Progression to NASH	Treatment
Obesity [34,35,36,37,38,39,40,41,42]	Increase prevalence	Increase risk	Weight loss, Bariatric Surgery
Insulin resistance [45,46]	Increase prevalence	Increase risk	Lifestyle modifications Pioglitazone may improve histological features of NASH. Limited evidence available on clinical utility of Glucagon like peptide 1 antagonists (GLP-1)
Hyperlipidemia [60,61,62,63,64,65,66,67,68,69,70]	Increase prevalence	Increase risk	Statins have shown to improve hepatic fibrosis. It can also reduce cardiovascular mortality
Intestinal Microbiota and oxidative stress [71,72,73,74]	Higher prevalence of small intestinal bacterial overgrowth in NAFLD.	Increase risk	Rifaximin has demonstrated benefit but further research is needed.Antioxidants like vitamin E have shown benefit in patients with NAFLD
Metabolic Syndrome [38,50,75]	Increase prevalence	Increase risk	Lifestyle modifications, statins, pioglitazone, weight loss.

**Table 4:** The NAFLD activity score (NAS) for the histopathologic diagnosis of nonalcoholic steatohepatitis (NASH) [84].

Histological Features	Extent	Score
Steatosis	Extent of involvement of parenchyma by steatosis	
	<5%	0
	5%–33%	1
	33%–66%	2
	>66%	3
Ballooning	No ballooned cells	0
	Few ballooned cells	1
	Many cells with ballooning	2
Lobular Inflammation	No inflammatory Foci per 200 Field	0
	<2 foci per 200 field	1
	2–4 foci per 200 field	2
	>4 foci per 200 field	3
NAS	Sum of steatosis + ballooning + lobular inflammation	
	Score 0–2	NASH unlikely
	Score 3–4	Borderline
	Score 5–8	Likely NASH

**Table 5:** CRN histological scoring system for grading of fibrosis in NAFLD [84].

Stage of Fibrosis	CRN Scoring System
0	No fibrosis
Stage 1 A	Mild perisinusoidal
Stage 1 B	Moderate perisinusoidal
Stage 1 C	Portal/periportal fibrosis
Stage 2	Perisinusoidal and portal/periportal fibrosis
Stage 3	Bridging fibrosis
Stage 4	Cirrhosis

**Table 6:** Noninvasive serologic tests for the diagnosis of NAFLD.

Serologic Test	Component of the Test	Clinical Utility
Aminotransferases [86]	ALT and AST	May be elevated in NAFLD patients
Cytokeratin-18 [87]	Cytokeratin-18	Elevated levels in NASH patients
AST/platelet ratio index (APRI) [88]	AST and platelets	Predicting fibrosis
NAFLD fibrosis score (NFS) [89,90]	Age, BMI, blood glucose levels, aminotransferase levels, platelet count, and albumin	Predicting advanced fibrosis and clinical outcomes in NAFLD patients
FIB-4 index [90,91]	Age, AST, ALT and platelet count	Predicting advanced fibrosis and clinical outcomes in NAFLD patients
FibroTest [92,93,94]	Age, sex, alpha-2-macroglobulin, haptoglobin, gamma globulin, apolipoprotein A1, gamma glutamyl transferase and total bilirubin levels	Predicting extent of fibrosis
ActiTest [93,94]	Age, sex, alpha-2-macroglobulin, haptoglobin, gamma globulin, apolipoprotein A1, gamma glutamyl transferase and total bilirubin and ALT levels	Predicting necroinflammatory activity
Enhanced Liver Fibrosis panel (ELF) [95,96]	Matrix metalloproteinase 1 (MMP-1), HA and amino-terminal propeptide of type III collagen level	Predicting extent of fibrosis
FibroSpect II [97]	Hyaluronic acid, tissue inhibitor of metalloproteinase-1 (TIMP-1), and alpha-2-macroglobulin.	Predicting extent of fibrosis

**Table 7:** Imaging modalities for the diagnosis of NAFLD.

Imaging Modality	Clinical Utility	Limitations
Ultrasound Abdomen [98]	Widely available and convenient Sensitivity and specificity for diagnosing fatty liver disease is 85% and 94% respectively	Operator dependent Lacks sensitivity in NAFLD patients with less than 30% steatosis on liver biopsy
CT abdomen [99]	Limited clinical utility in diagnosing NAFLD	Radiation hazard, introduces contrast-related risks, has low sensitivity for hepatic fat mapping
Magnetic resonance spectroscopy [99,100,101]	Allows for quantification of hepatic fat	Not available on all scanner
Transient Elastography (Fibroscan) [102,103]	Sensitivity of 88% with a negative predictive value of 90% in detecting advanced fibrosis	Presence of ascites, obese patients or presence of acute inflammation
Magnetic Resonance elastography [104,105]	Sensitivity of 86% and specificity of 91% for diagnosing advanced fibrosis	Limited availability, expertise to interpret the results, cost of the procedure, presence of metal implants, patient’s size and claustrophobia
Shear wave elastography (SWE) [106]	Sensitivity of 90% and the specificity of 88% in detecting advanced fibrosis	Limited evidence available current and needs further research on its clinical utility
